# Supplementary material for: A Comprehensive Assessment of Ultraviolet-Radiation-Induced Mutations in Flammulina filiformis Using Whole-Genome Resequencing
Source: J Fungi (Basel). 2024 Mar 20;10(3):228. doi: 10.3390/jof10030228 (PMC10971301; doi:10.3390/jof10030228)
Supplement: Supplementary file 1 [file jof-10-00228-s001.zip › Supplementary Material S8/KEGG annotation/out/64381550635650.os/KO/out_map/map05134.html]

KEGG PATHWAY: Legionellosis - Reference pathway


|  |  |
| --- | --- |
| **Legionellosis - Reference pathway** |  |

[
Pathway menu
| Organism menu
| Pathway entry
| Show description
| User data mapping
]

Legionellosis is a potentially fatal infectious disease caused by the bacterium Legionella pneumophila and other legionella species. Two distinct clinical and epidemiological syndromes are associated with Legionella species: Legionnaires' disease is the more severe form of the infection, which may involve pneumonia, and Pontiac fever is a milder respiratory illness.
The pathogenesis of L. pneumophila is derived from its growth within lung macrophages. One of the L. pneumophila's type IV secretion systems, the Dot/Icm secretion system, is of critical importance for its ability to replicate and to cause disease. The Dot/Icm substrates modulate multiple host cell processes and in particular, redirect trafficking of the L. pneumophila phagosome and mediate its conversion into an ER-derived organelle competent for intracellular bacterial replication. L. pneumophila also manipulates host cell death and survival pathways in a way that allows continued intracellular replication.

|  |  |
| --- | --- |
| Reference pathway | 100% |
